# Functional biases in attentional templates from associative memory

**DOI:** 10.1167/jov.20.13.7

**Published:** 2020-12-09

**Authors:** Sage E. P. Boettcher, Freek van Ede, Anna C. Nobre

**Affiliations:** 1 Department of Experimental Psychology, University of Oxford, Oxford, UK; 2 Oxford Centre for Human Brain Activity, Wellcome Centre for Integrative Neuroimaging, Department of Psychiatry, University of Oxford, Oxford, UK; 3 Oxford Centre for Human Brain Activity, Wellcome Centre for Integrative Neuroimaging, Department of Psychiatry, University of Oxford, Oxford, UK; 4 Institute for Brain and Behavior Amsterdam, Department of Experimental and Applied Psychology, Vrije Universiteit Amsterdam, The Netherlands; 5 Department of Experimental Psychology, University of Oxford, Oxford, UK; 6 Oxford Centre for Human Brain Activity, Wellcome Centre for Integrative Neuroimaging, Department of Psychiatry, University of Oxford, Oxford, UK

**Keywords:** target template, long-term memory, working memory, paired associate, proactive, prediction, preparation, vision, feature weighting

## Abstract

In everyday life, attentional templates—which facilitate the perception of task-relevant sensory inputs—are often based on associations in long-term memory. We ask whether templates retrieved from memory are necessarily faithful reproductions of the encoded information or if associative-memory templates can be functionally adapted after retrieval in service of current task demands. Participants learned associations between four shapes and four colored gratings, each with a characteristic combination of color (green or pink) and orientation (left or right tilt). On each trial, observers saw one shape followed by a grating and indicated whether the pair matched the learned shape-grating association. Across experimental blocks, we manipulated the types of nonmatch (lure) gratings most often presented. In some blocks the lures were most likely to differ in color but not tilt, whereas in other blocks this was reversed. If participants functionally adapt the retrieved template such that the distinguishing information between lures and targets is prioritized, then they should overemphasize the most commonly diagnostic feature dimension within the template. We found evidence for this in the behavioral responses to the lures: participants were more accurate and faster when responding to common versus rare lures, as predicted by the functional—but not the strictly veridical—template hypothesis. This shows that templates retrieved from memory can be functionally biased to optimize task performance in a flexible, context-dependent, manner.

## Introduction

Attentional templates are the mental representations that we use to facilitate the detection and identification of task-relevant sensory inputs (Bundesen, Habekost, & Kyllingsbæk, 2005; Carlisle, Arita, Pardo, & Woodman, 2011; Chelazzi, Duncan, Miller, & Desimone, 1998; Chelazzi, Miller, Duncan, & Desimone, 1993; Desimone & Duncan, 1995; Duncan & Humphreys, 1989; Geng & Witkowski, 2019; Kok, Mostert, & De Lange, 2017; Treue & Martinez Trujillo, 1999). A common intuition is that the attentional template is a replica of the anticipated target. However, templates may not always be fully specified or strictly veridical. For example, we are able to search for an item at the category level—that is, without knowing the exact features of the target (Cunningham & Wolfe, 2014; Peelen & Kastner, 2011)—and objects that are functionally related to the target template can guide visual search (Boettcher, Draschkow, Dienhart, & Võ, 2018).

Recently, an alternative and more flexible view of attentional templates is emerging, with their functional nature prevailing over their mimetic quality. Rather than a faithful reproduction of the anticipated target, a template may be adapted to optimize perceptual performance within a current task context. A striking recent demonstration comes from work by Yu and Geng (2019). Whereas templates resembled targets when searching for a target color (orange) in arrays with an equal likelihood of distractors from either side of the color space (yellower and redder), templates became distorted when distractors were all drawn from one side of the color space (e.g., yellower). In this case, when targets and distractors were linearly separable, the attentional template was asymmetrically sharpened and repelled away from distractors (becoming redder). In such a functional framework, certain template features may be overemphasized and/or distorted in service of anticipated task requirements (for a recent review see Geng & Witkowski, 2019).

Our aim was to investigate whether the functional properties of attentional templates also generalizes to templates retrieved from associative memory (Boettcher, Stokes, Nobre, & van Ede, 2020). In typical laboratory studies of attentional templates, the template is explicitly provided before the start of a trial or block of trials (Carlisle et al., 2011; Chelazzi et al., 1993; Lee & Geng, 2019; Navalpakkam & Itti, 2007; van Driel, Gunseli, Meeter, & Olivers, 2017; Yu & Geng, 2019). However, in the real world, attentional templates are more often derived from learned associations between stimuli, such that one stimulus (A) predicts another (B) (Boettcher et al., 2020; Higuchi & Miyashita, 1996; Hutchinson & Turk-Browne, 2012; Kok et al., 2017; Stokes, Thompson, Nobre, & Duncan, 2009). Can the template for a stimulus B—retrieved on the basis of its long-term memory association to stimulus A—*also* be functionally biased in service of anticipated task demands?

There are at least two reasons why templates retrieved from long-term memory may not be as adaptable as those explicitly presented. First, unlike functional biases of templates that are explicitly presented, templates retrieved from memory cannot benefit from differences at the encoding stage (Reeder, Hanke, & Pollmann, 2017; Serences, Ester, Vogel, & Awh, 2009). Second, unlike forms of adaptation that may gradually develop when repeatedly using the same template for many trials in a row (as in, e.g., Navalpakkam & Itti, 2007; Yu & Geng, 2019), the same associative memory template may require distinct adaptations at each instance of retrieval depending on the current context. As such, these templates may not benefit from potential functional biases at the level of the stored memory trace, but rather from biases that develop during or after their retrieval. Therefore an important open question remains regarding whether templates that are retrieved from associative memory can also be functionally biased to optimally serve behavior.

Here we developed a task in which observers retrieved templates from memory. Between relatively short blocks lasting only a few minutes, one or the other visual feature of the template (color or tilt) was made more informative by increasing the proportion of nonmatch (lure) items that differed along one of the features. For example, if the majority of lures would share the same color as the target, template color would become less informative, and tilt might be emphasized instead. Here, we studied this in a context in which the associations varied randomly throughout the block (to reduce the potential influence of priming or the repeated use of a single template that is not necessarily being retrieved from long-term memory). If templates are inflexible and veridical, their constituent features should have equal weightings, predicting consistent behavior regardless of the lure type. In contrast, if templates retrieved from memory can be functionally adapted to overemphasize the most useful feature dimension, we should find evidence for this in the pattern of behavioral response as a function of block.

We hypothesized that templates retrieved from long-term memory can become functionally biased, and that may occur similarly for distinct feature dimensions. At the same time, because the prioritization of different visual feature dimension may (at least under certain circumstances) be asymmetrical (e.g., Biderman, Biderman, Zivony, & Lamy, 2017; Geng, Di Quattro, & Helm, 2017; Lee & Geng, 2019; Niklaus, Nobre, & van Ede, 2017), we remained open to the possibility to find differences in functional biases of visual color and tilt information (the two feature dimensions that we evaluated here).

## Methods

### Participants

Twenty-two participants took part in the Experiment. Two participants failed to complete the experiment due to problems with the testing computer. The remaining 20 participants (13 female, 1 left handed) were between 21-35 years old with an average age of 24.6. All Participants had normal or corrected-to-normal vision, provided written consent prior to participation, and were compensated at a rate of £10 per hour.

### Task and procedure

All experimental procedures were reviewed and approved by the Central University Research Ethics Committee of the University of Oxford. Participants completed the experiment in a group testing room with a capacity of 20 people, although no more than 12 people were tested at one time. Participants each sat approximately 50 cm from the monitor (Dell U2312HM Monitor, 1920 × 1080 resolution; refresh rate 60 Hz). The experimental script was generated using the Psychophysics Toolbox (Brainard, 1997) on MATLAB (version 2014b; The MathWorks Inc., Natick, MA, USA).

**Figure 1.**
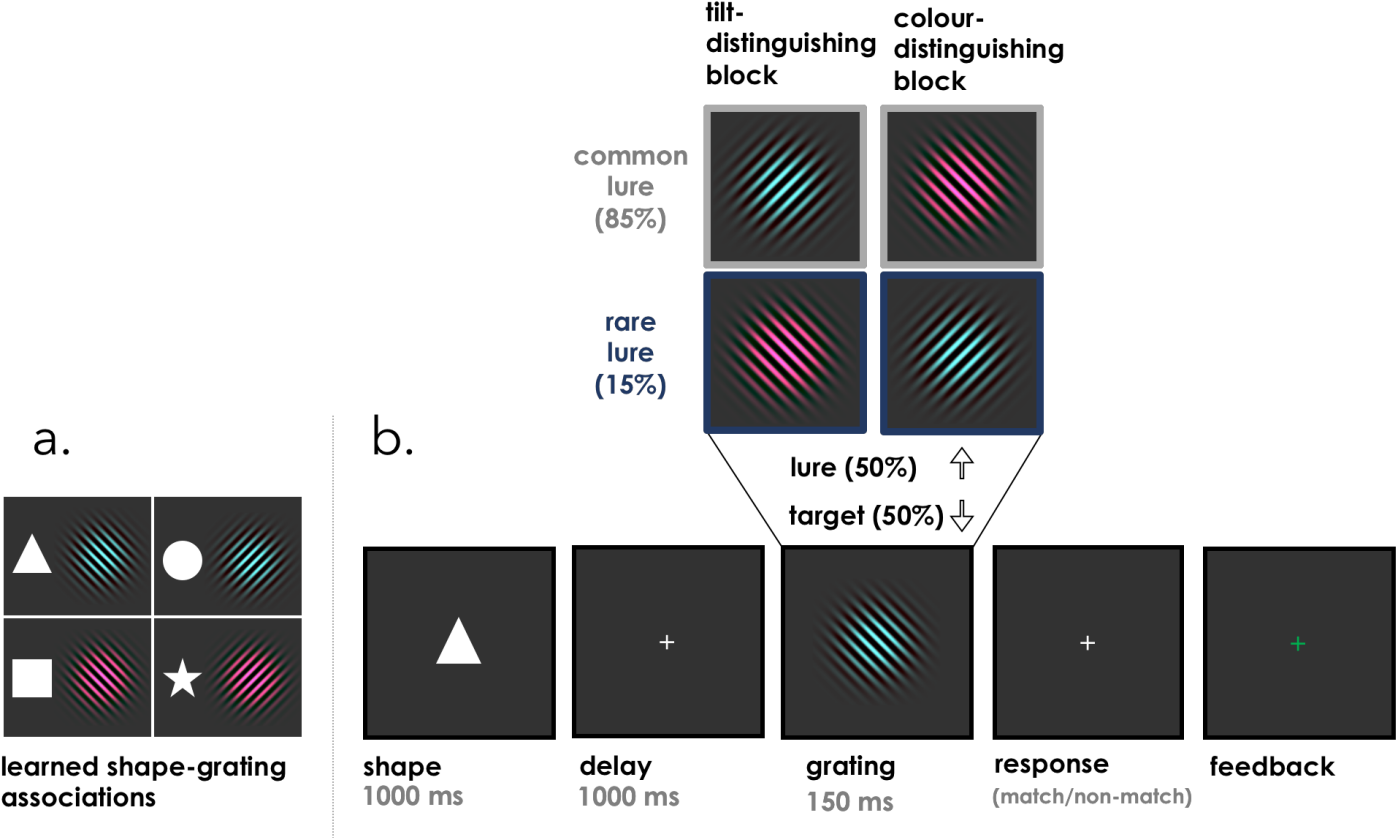
Trial schematic of experiment. (a) In the Learning phase, each observer learned to associate four shapes to four gratings. These pairings were randomized across participants. (b) In the test phase, each trial began with the presentation of one of the 4 shapes followed by a blank and then a grating. Observers indicated whether the shape-grating matched the association held in memory in both color and tilt. On non-match (lure) trials, observers could be presented with a common or rare lure, such that lures were more likely to have a different color (“color-distinguishing block”) or a different tilt (“tilt-distinguishing block”). Note that if a different shape was presented—all shapes were equally likely—the roles of the gratings would also change. For example, if a star was presented, then the expected grating would be a pink-right tilted target, and the lures would change accordingly.

Prior to the main experimental task, participants took part in a learning phase in which they learned the relationship between four distinct shapes (square, star, circle, and triangle) and four gratings (Figure 1a; shape-grating pairings were randomized across participants). The four gratings each had a unique combination of color (green or pink; RGB values: [46, 142, 141], [246, 37, 113]) and tilt (left or right, at an angle of ± 45°). On each trial, observers saw a central shape (1.4°) followed by a central grating (9.1°) and indicated whether the pair matched the learned shape-grating association. Shapes appeared for 1000 ms followed by a 1000-ms delay. The grating then appeared for 150 ms. Observers were required to respond within 1500 ms with the “j” key (right hand response) if they believed the stimulus matched the learned shape-grating association in memory (in both color and tilt) and the “f” key (left hand response) if the grating did not match the learned association (because either the color or the tilt was different). On 50% of the trials a match grating was presented whereas on the other 50% of the trials a nonmatching lure stimulus was presented. Nonmatching (lure) stimuli matched the learned association in one dimension but not the other (Figure 1b). In the learning phase, participants were required to complete a practice block of 30 trials with above 80% accuracy before moving on to the test phase (main verification task) that followed the same procedure, but where we manipulated the type of non-match gratings across blocks. These pairings were further reinforced during the experiment in the form feedback after each trial.

The main verification task consisted of 30 blocks with 30 trials in each block—with each block lasting approximately three minutes. The central experimental manipulation concerned how informative each feature dimension was for discriminating targets from lures by changing the probability that the lure shared either color or tilt with the target across blocks. Specifically, in half of the blocks the lure was most likely to differ from the target in color but not tilt (color-distinguishing blocks), whereas in the other blocks this pattern was reversed (tilt-distinguishing blocks). Block type was randomly ordered across 15 pairs (e.g., AB BA AB …). We will refer to these nonmatch gratings as *common* and *rare* lures. Common lures occurred on 85% of lure trials whereas rare lures occurred on 15% of lure trials. For example, in a color-distinguishing block, in 85% of the lure trials the grating differed from the expected target in color but not tilt (common), whereas in the remaining 15% of the lure trials the grating differed from the expected target in tilt but not color (rare). If templates retrieved from associative memory are veridical and therefore both features are weighed equally, then we should see no effect of lure type. However, if the attentional template for the upcoming stimulus is adapted such that the distinguishing feature is emphasized, then the common lure will be more distinct from the template compared with the rare lure. It follows that participants should be more accurate and faster to reject common lures compared with rare lures.

After observers responded, or if they were too slow with their response (> 1500 ms; this occurred in less than 1% of trials across all participants), feedback was provided in the form of a color change in the fixation cross—green for correct response, red for incorrect responses, and orange on trials in which a response was not provided in time.

All feature values were equally probable within each block, and participants were never tasked with repeatedly searching for a specific feature value throughout a given block. What defined the target, the common lure, and the rare lure was solely and completely dependent on the preceding shape cue. Because the shape cues were randomly interleaved across trials, participants were required to upload a fresh attentional template on each trial. As such, there is no possibility that low-level adaptation or short-term priming could explain any differences in performance in the different block types.

### Statistical analysis

Because the critical manipulation (of block type) regarded the type of lures that could occur, we restricted our analyses to lure trials. Generalized linear mixed-effects models (GLMMs) with a binomial distribution were used to analyses the percentage false alarms (FAs), and linear mixed-effects models (LMMs) to analyses reaction times (RTs) (correct rejection trials only) in a procedure similar to the approach described in Helbing, Draschkow, and Võ (2020) and Draschkow and Võ (2017). Trials in which the response times were faster than 200 ms or greater than 3 standard deviations from the participant's mean were discarded from the analysis. This resulted in an average loss of 1.4% ± 0.19% (*m* ± standard error of the mean [*SEM*]) of trials. These analyses were run using the lme4 package (version 1.1-17; Bates et al.*,* 2015). We used mixed-effects models because they hold multiple benefits over a more traditional approach to analysis of variance. Importantly for the current study, these approaches are more reliable in unbalanced designs when different conditions have different trial numbers—for example, common versus rare lures (Baayen, Davidson, & Bates, 2008). All GLMMs and LMMs were fitted with the maximum likelihood criterion. For the GLMMs, where we report regression coefficients *β* with the z statistic and use a two-tailed 5% error criterion for significance, the *p* values for the binary accuracy variable are based on asymptotic Wald tests. For the LMMs, we report *β* with the *t* statistic and apply a two-tailed criterion corresponding to a 5% error criterion for significance. The *p* values were calculated with Satterthwaite's degrees of freedom method using the lmerTest package (version 3.1-0; Kuznetsova, Brockhoff, & Christensen, 2017). Pairwise tests after significant interactions were further investigated using the lsmeans package (Lenth, 2016) with Tukey post-hoc correction.

In this experiment there were two main independent variables of interest: lure type (common vs. rare) and, block type (color-distinguishing and tilt-distinguishing). The comparisons were modelled using sum contrasts, in which the grand mean of the dependent measure served as the intercept. For binary responses such as FAs in the GLMM approach, the coefficients are represented by logits. We began each model with a maximal random-effects structure (Barr, Levy, Scheepers, & Tily, 2013) that included intercepts for each participant, as well as by-participant slopes for the effects of lure type and block type. Full models such as these often fail to converge or lead to overparameterization (Bates, Kliegl, Vasishth, & Baayen, 2015). Therefore we used a principal component analysis (PCA) of the random-effects variance-covariance estimates to identify overparameterization for each fitted model and removed random slopes that were not supported by the PCA (i.e., did not explain significant variance in the model) and did not contribute significantly to the goodness of fit in a likelihood ratio (LR) test (Bates, Kliegl, et al., 2015). For both the GLMM and LMMs, this resulted in the removal of the by-subject slopes for lure type from the random effects, and therefore the random-effects structures for the optimal models included the subject intercepts, as well as by-subject slopes for block type. Further details regarding the models and model comparisons can be found in the analysis script. Analysis scripts and data can be found here, https://osf.io/x7u4q/.

To make sure the results did not depend on the chosen approach, we also conducted traditional repeated-measures analyses of variance for both FA and RT. These showed equivalent results and can also be found in the analysis script provided on OSF on acceptance. The ggplot2 package (version 3.1.0; Wickham, 2009) was used for plotting results. Furthermore, where relevant, the within-subject standard error of the mean was calculated from normalized data using the approach from Morey (2008).

## Results

**Figure 2.**
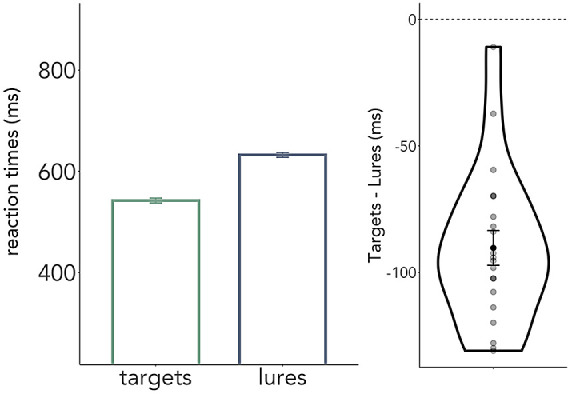
Target templates drive behavior. Observers were quicker to react to targets compared to lures, indicating that the target representation was driving behavior. The right panel shows the difference in RT between targets and lures, with individual subjects’ differences plotted as gray dots.

Observers correctly identified the target in 93.5% ± 1.1 (*m* ± *SEM*) of match trials, indicating that they had properly learned the shape-gratings associations, and were able to complete the task to a high standard. Moreover, when considering only correct trials, observers were faster to affirm targets than to reject lures (Figure 2) (*β* = 0.04, *SE* = 0.03, *t* = 13.08, *p* < 0.001). This suggests they were (primarily) relying on a positive template of the target rather than a negative template of the potential lures, which is also theoretically possible (Arita, Carlisle, & Woodman, 2012; Conci, Deichsel, Müller, & Töllner, 2019; Moher & Egeth, 2012).

**Figure 3.**
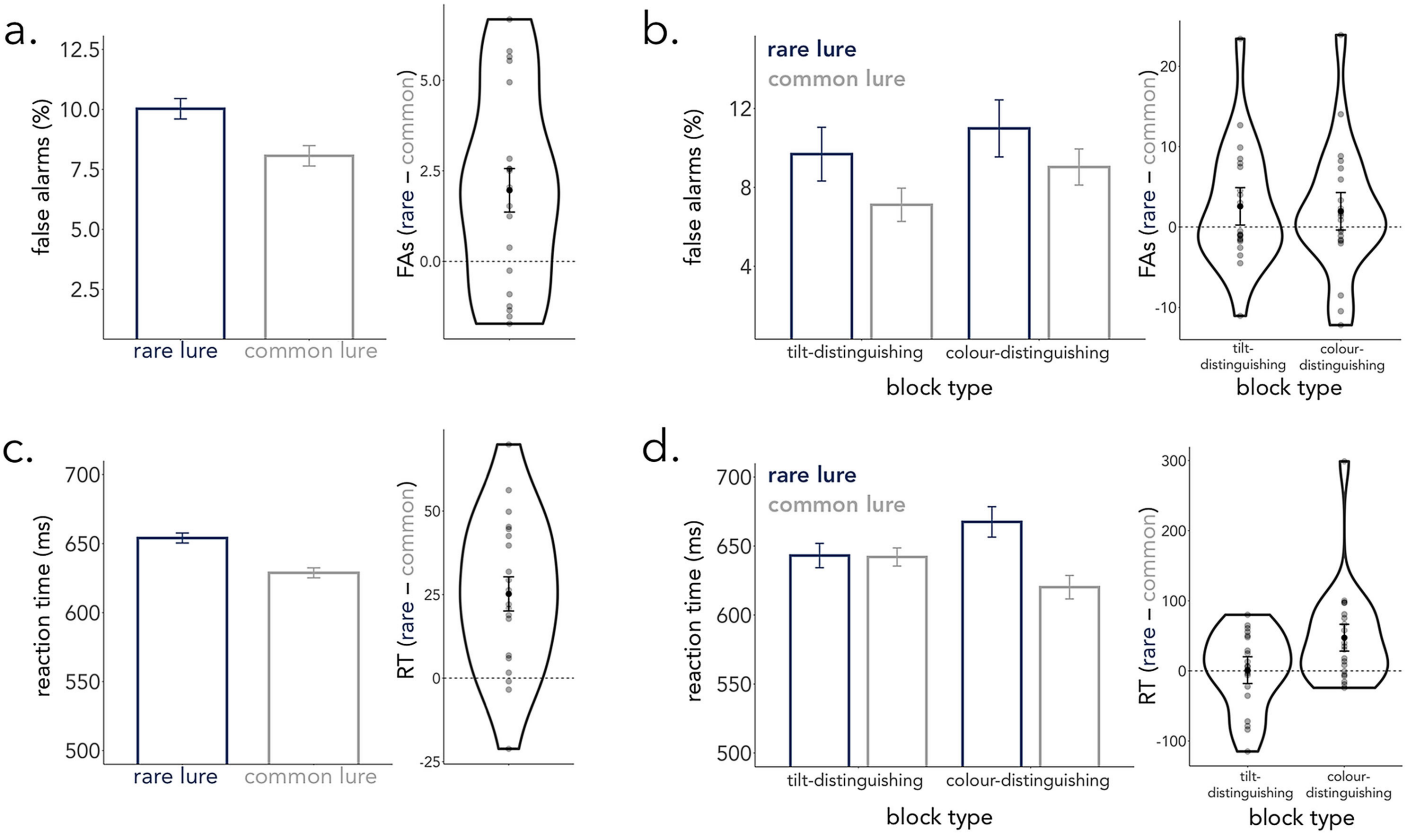
Functional adaptation in attentional templates retrieved from long-term memory associations. (a) Observers made fewer false alarms to common lures than rare lures. The right panel (violin plot) shows the effect of lure type on false alarms (rare—common) for individual observers, represented as dots. The average effect is plotted as a black dot and error bars represent the within-subject standard error (this is true for all panels). (b) The effect of lure type was similar across block types. (c) Participants were quicker to correctly reject a common lure compared to a rare lure. The effect of lure type is plotted for individual observers in the right panel. (d) The effect of lure type on reaction times was only significant in the color-distinguishing block. That is, observers were significantly slower to respond to lures that shared their color with the target template when color was usually the distinguishing feature.

Having established that observers were able to do the task to a high standard and were likely using positive templates we now shift our focus to participants’ responses to the nonmatch (lure) stimuli, because these were the subject of our primary experimental manipulation. If participants are using a functional template biased by which feature dimension is most informative in the current context (here manipulated at the level of blocks), then this template should become optimized for rejecting the type of lure that was more common in this context (block). In line with this hypothesis, we found that observers were less likely to erroneously identify a common (vs. a rare) lure as a target (i.e., false alarm) (Figure 3a) (*β* = 0.13, *SE* = 0.05, *z* = 2.52, *p* = 0.01; *rare lures*: 10.02% ± 0.42%; *common lures*: 8.06% ± 0.42%). This pattern was similarly present for both visual features (Figure 3b): we found no effect of block type (*β* = −0.03, *SE* = 0.07, *z* = −0.50, *p* = 0.62) and no interaction between lure type and block type (*β* = 0.03, *SE* = 0.05, *z* = 0.56, *p* = 0.57) (Figure 3b).

Analysis of reaction times yielded complementary evidence. Observers were faster to reject common lures (628 ms ± 3.62) compared to rare lures (654 ms ± 3.62) (Figure 3c) (*β* = 0.02, *SE* = 0.003, *t* = 4.23, *p* < 0.001). We found no effect of block type (*β* = 0.001, *SE* = 0.005, *t* = 0.233, *p* = 0.82). However, for RT, block type and lure type did show a significant interaction (*β* = −0.008, *SE* = 0.002, *t* = −2.93, *p* = 0.003) (Figure 3d). Post-hoc comparisons revealed that there was no significant effect of lure type on RT in the tilt-distinguishing block (*β* = 0.0072, *SE* = 0.0078, *z* = 0.918, *p* = 0.79), although there was a significant effect of lure type in the color-distinguishing block (*β* = 0.0398, *SE* = 0.0078, *z* = 5.06, *p* < 0.001). That is, observers were faster in rejecting common lures that differed from the template in color but not tilt (as opposed to tilt but not color). This asymmetry is possibly related to other findings that have reported similar asymmetries between the prioritization of different feature dimensions (Biderman, Biderman, Zivony, & Lamy, 2017; Geng, Di Quattro, & Helm, 2017; Lee & Geng, 2019; Niklaus, Nobre, & van Ede, 2017), although we note that this asymmetry that we observed in RT was not similarly present in the pattern of false alarms.

In a secondary analysis we assessed whether the lure effect on false alarms and reaction times was affected by time in block—here operationalized as first and second half of a block (Supplementary Figure S1). Time in block had no effect on false alarms (*β* = −0.07, *SE* = 0.05, *z* = −1.46, *p* = 0.14) and did not significantly interact with the lure type (*β* = 0.02, *SE* = 0.05, *z* = 0.30, *p* = 0.76) indicating that the effects of the lure were similarly present in the first and second half of a block (Supplementary Figure S1a). This was also true for reaction times. That is, we found no main effect of time in block on reaction times (*β* = 0.002, *SE* = 0.002, *t* = 0.57, *p* = 0.566), and this factor did not interact significantly with lure type (*β* = 0.002, *SE* = 0.002, *t* = .60, *p* = 0.55) (Supplementary Figure S1b).

## Discussion

We provide clear evidence for a functional interpretation of attentional templates based on associative memories. When a common lure was presented, participants were less likely to confuse these for targets (decreased false alarms) and were quicker to correctly reject these gratings, compared to when a rare lure was presented. This indicates that the template for the upcoming stimulus had been adapted away from the common type of lures in a block, such that the most diagnostic and therefore informative feature dimension—for distinguishing between targets and lures—was prioritized in the template. We found this despite the fact that the relevant feature dimension varied block wise (with blocks lasting only a few minutes). Moreover, within each block, the feature values of the relevant attentional templates themselves changed from trial to trial, showing that the prolonged and repeated use of the same template is not necessary for a template to become adapted to the current context. Additionally, it is relevant to note that we found functional biases, even though observers completed the task with high accuracy and the features were easily discriminable.

Our results complement and extend previous work on optimizing attentional templates to current task demands, which had focused primarily on templates that were explicitly provided at the beginning of a trial of block of trials, such as in traditional working memory or visual search tasks. For example, it has been shown that observers can selectively encode only the relevant feature of an object (Reeder et al., 2017; Serences et al., 2009) as well as update working memory content after encoding, such that the representation is biased towards the most relevant feature dimension (Hajonides, van Ede, Stokes, & Nobre, 2020; Niklaus et al., 2017; Park, Sy, Hong, & Tong, 2017). Moreover, when targets and distractors are linearly discriminable within a certain feature dimension, target representations are biased away from the distractors (Bauer, Jolicoeur, & Cowan, 1996; Becker, Folk, & Remington, 2010; Geng et al., 2017; Geng & Witkowski, 2019; Hodsoll & Humphreys, 2001; Navalpakkam & Itti, 2007; Yu & Geng, 2019). Building on this previous work, we provide evidence that attentional templates retrieved from associative memory are also adaptable to a particular context—an important advance because real-life attentional templates are often based on associations in long-term memory.

With the current design we attempted to minimize other potential sources of behavioral biases. For example, by introducing four distinct cues associated with four distinct gratings, the chance that a particular cue-grating repeats from one trial to the next is relatively low. As such, we hoped to limit the effects of inter-trial priming (Kristjánsson & Campana, 2010; Maljkovic & Nakayama, 2000; Meeter & Olivers, 2006). Even so, it is still possible that priming may interact with the proactive biasing we find here. For example, if a particular cue repeats, observers may not need to retrieve the template from long-term memory, because it is likely already available in short-term store. This may exaggerate the difference between common and rare lures. Future work could directly manipulate the number of repetitions within a block to understand how template biasing and priming interact.

Our results show that the attentional template itself—not only a spatial priority map—may be functionally biased toward the relevant feature dimension. Information used to guide spatial attention during visual search—attentional priority maps (Itti & Koch, 2001; Wolfe, 1994)—and the template information to which we match incoming sensory information, are not often explicitly distinguished within the literature (Wolfe, 2020). Within the context of visual search, “dimension-weighting” has been argued to interact with the attentional priority map to guide spatial attention to a target (Liesefeld & Müller, 2019; Müller, Heller, & Ziegler, 1995). In the current work, we used a task other than visual search, in which a single stimulus was presented centrally, and therefore did not require shifts in spatial attention, nor a search among competing distractors. This allowed us to isolate the influence of our experimental manipulation of context, at the level of the template.

The current work focused on biases within the attentional template representing the target. Recently, there has been evidence that negative templates—that is, lure information that should be avoided—can also help guide attention (Arita et al., 2012; Conci et al., 2019; Moher & Egeth, 2012). In the current experiment, the target information was always known while the lure could have been one of two potential items. In line with this, our results show that observers were faster to respond to targets compared to lures, indicating that they are likely responding on the basis of a (positive) target template. Whether negative templates remain subject to the same potential biases as positive templates remains an interesting topic for future research.

The current task raises an interesting question at which point the template becomes functionally biased. First, it remains possible that the representation is in fact biased within long-term memory to overemphasize a particular feature dimension and then this representation is biased back with the change of block. In our design, we discouraged this possibility by using relatively short blocks—each lasting only a few minutes—so that observers would be required to adapt quickly to the new surroundings. Moreover, a secondary analysis revealed no difference in the biasing effect in the first and second halves of each blocks. Nevertheless, we cannot fully rule out that longer-term biases in the stored template may have also contributed to (at least part of) the reported effects. In future work if will be of interest to investigate the relative contributions of biases that gradually form in long-term memory versus those that are instantiated after retrieval.

Even if we assume that participants relied on veridical and stable cue-target representations stored in long-term memory, there are still two viable stages at which the observed functional adaptation may occur: at the retrieval stage (retrieving primarily the most diagnostic feature), or later, in the cue-target interval, after the template has been brought into working memory (Atkinson & Shiffrin, 1968; Fukuda & Woodman, 2017), where it may be particularly moldable (D'Esposito & Postle, 2015; Griffin & Nobre, 2003; Niklaus et al., 2017; Rerko & Oberauer, 2013). Regardless of the answer to this interesting, but also challenging, question, the current results demonstrate that attentional templates retrieved from long-term memory associations are flexible, functionally adapt to the context in which they are retrieved, and are expected to be used.

## Supplementary Material

Supplement 1
