# Supplementary material for: Functional biases in attentional templates from associative memory
Source: J Vis. 2020 Dec 9;20(13):7. doi: 10.1167/jov.20.13.7 (PMC7729124; doi:10.1167/jov.20.13.7)
Supplement: Supplement 1 [file jovi-20-13-7_s001.docx]

**Supplementary Figure**

### **
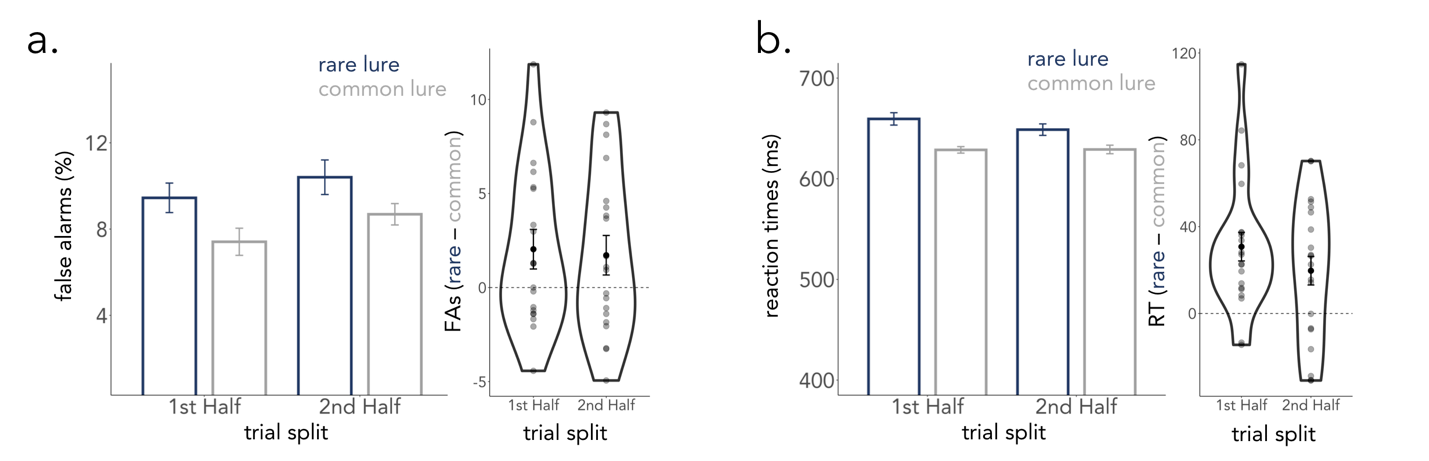
Supplementary Figure 1. Functional adaptation in attentional templates is not modulated by time in block.** We split the trials into those that occurred in the first half of a block (1^st^ Half) and those that occurred in the second half of the block (2^nd^ Half). (a) The effect of lures on False Alarms is plotted separately for those that occurred in the first half of a block and those that occurred in the second half. We found no effect of the trial split and this factor did not significantly interact with lure type, indicating that the lure effect occurred early in a trial and was persistent though out. b) This was also true for reaction times. Again, we found no main effect of trial split on reaction times and this did not significantly interact with lure type.
